# The impact of export shocks on child health: evidence from China

**DOI:** 10.3389/fpubh.2025.1593356

**Published:** 2025-08-14

**Authors:** Yu-Ting Zhang, Oksana Vladimirovna Mashevskaya, Xiu-Zhi Wang

**Affiliations:** ^1^Faculty of Economics, Belarusian State University, Minsk, Belarus; ^2^School of Economics and Management, Nanchang Hangkong University, Nanchang, China

**Keywords:** export shocks, child health, morbidity, Bartik-IV, trade liberalization

## Abstract

**Background:**

Amidst sluggish global economic growth and rising anti-globalization sentiments, it has become imperative to reassess the benefits and hidden costs of globalization. Simultaneously, with the fading of the “demographic dividend” and the intensification of population aging, understanding children’s health and its underlying determinants is crucial for sustainable socio-economic development. China provides an ideal case for examining these issues.

**Methods:**

Using data from the China Health and Nutrition Survey (CHNS) and UN Comtrade Database, this study constructs a four-period unbalanced panel and empirically investigates the impact of export expansion on children’s health in China during the country’s trade liberalization process. To address potential endogeneity concerns, we employ a Bartik-IV model to construct an export shock variable and identify the effect of trade exposure on child morbidity.

**Results:**

Regression estimates reveal that export shocks are significantly associated with improved child health. Specifically, a one-standard-deviation increase in export exposure reduces the probability of child morbidity by 14 percentage points. Mediation analysis identifies four key channels: parental fixed income, employment rates, work intensity, and left-behind child status. Export-induced increases in parental wages and paternal employment are linked to better child health, while paternal work intensity also shows a protective effect. In contrast, increased maternal work intensity may worsen child outcomes. Export shocks also raise the incidence of left-behind children, with differing effects by gender. Heterogeneity analysis reveals that health gains from trade are concentrated among boys, urban children, and those in western China.

**Conclusion:**

Trade liberalization can yield important health benefits for children, but these gains are not evenly distributed. Policies should aim to strengthen positive mechanisms such as stable parental employment and income, while also addressing potential risks associated with increased maternal labor demands and child separation. Particular attention should be paid to left-behind girls and to children living in rural areas of central and eastern China. Expanding investment in public health infrastructure, strengthening social protection systems, and ensuring equitable access to education are essential for making trade-driven growth conducive to inclusive and sustainable improvements in child health.

## 1 Introduction

Economic globalization has played a pivotal role in facilitating international division of labor, enhancing global connectivity, and driving economic growth (1). However, it has also been accompanied by challenges such as poverty (2), conflict (3), inequality (4, 5), and environmental pollution (6–8). The 2008 global financial crisis marked a turning point, as the world economy failed to achieve the expected recovery and instead fell into prolonged economic stagnation and structural downturns. Protectionist trade policies have escalated, regional trade and investment agreements have become increasingly fragmented, and populist, conservative, and isolationist sentiments have gained momentum. A striking example of unilateral trade protectionism emerged in 2020 when the Trump administration placed 58 Chinese and 45 Russian companies on the export control entity list, triggering widespread economic and political repercussions.^1^ This wave of “deglobalization” has compelled a reassessment of globalization’s benefits as well as its hidden costs. Given its distinct economic landscape and deep integration into global trade, China serves as an ideal case for exploring these dynamics.

**Figure 1:**
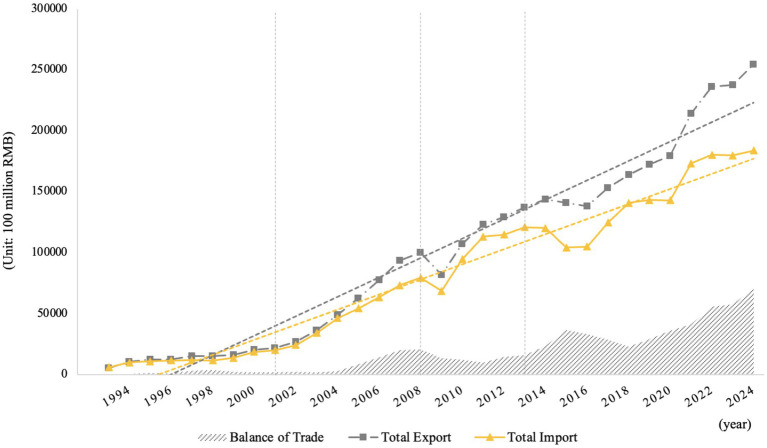
Changes in China’s import and export trade volume, 1993–2024. Source: compiled by the authors.

**Figure 2:**
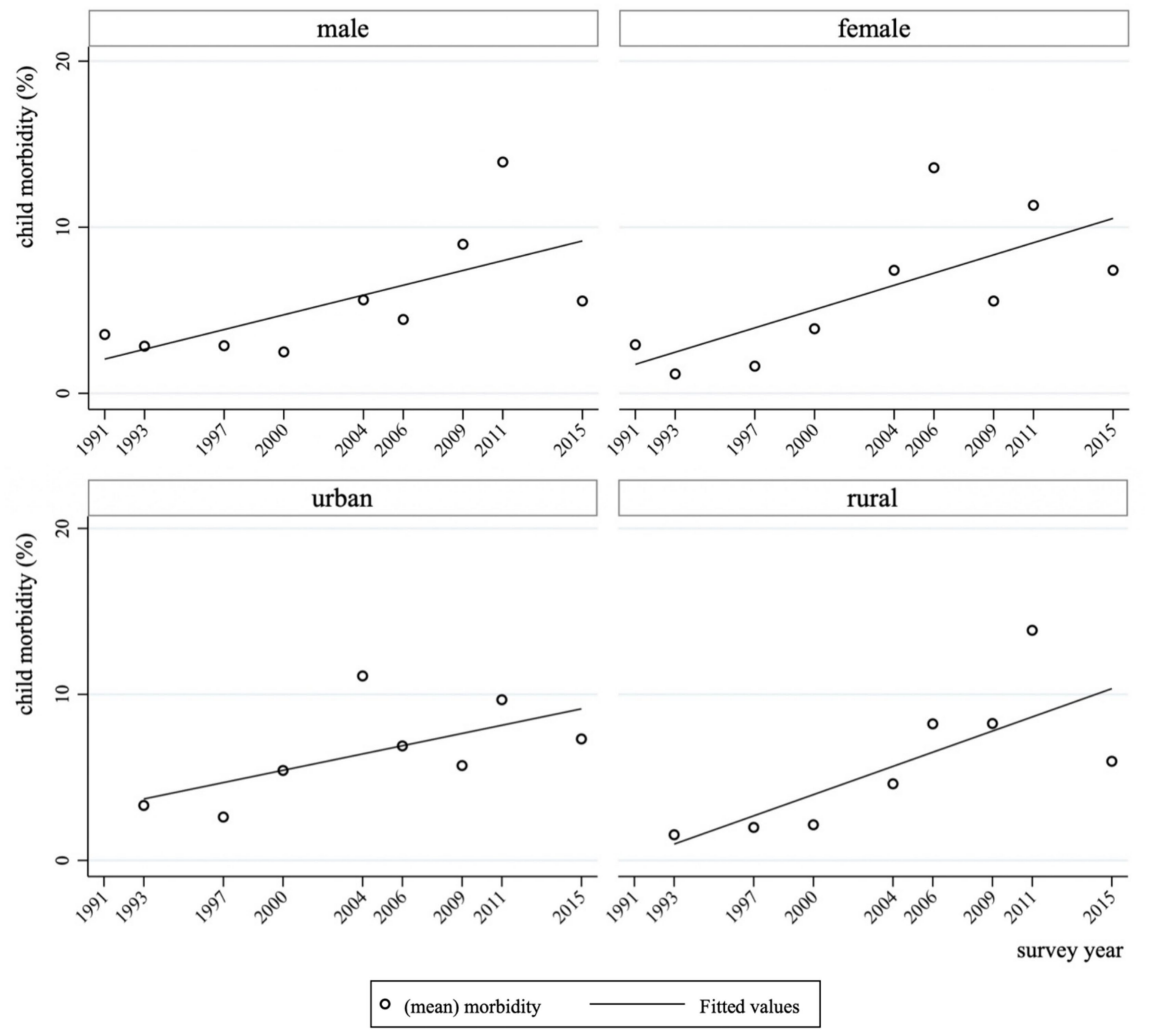
Trends in child morbidity by gender and household registration in China, 1991–2015. Source: compiled by the authors according to China Health and Nutrition Survey.

Since China’s accession to the World Trade Organization (WTO) in 2001, the volume of import and export trade in China has taken off rapidly, and the trade surplus has grown significantly^2^ (see Figure 1). Meanwhile, child morbidity has continued to increase in each region of China. Data show that the morbidity of children in both urban and rural areas in China has continued to rise since 1991–2015 (see Figure 2). Children’s health is intrinsically valuable as an end in itself (9), and child health is an important source of long-term economic growth and long-term social sustainability. From the perspective of micro-individuals, children’s health is closely linked to their health as adults, the level of education they can obtain, their productivity and their income (10, 11); from the perspective of macro-individuals, China is now facing a demographic transition during which the demographic dividend has gradually receded, and it is difficult to reverse this trend in the short term. Children’s health has a direct impact on the quality of the labor force in the future and even on the overall quality of the population. If trade seriously undermines children’s health, it is unlikely to contribute to long-term economic growth. It is of great practical significance to explore the relationship between trade liberalization and children’s health to promote sustainable economic and trade development.

To summarize, this study investigates the impact of export expansion on child health in the context of China’s trade liberalization. First, we construct a micro-level panel dataset by combining data from the China Health and Nutrition Survey (CHNS), the UN Comtrade Database, and the 0.95‰ Sample Survey of China’s 2000 Population Census. Second, to mitigate endogeneity concerns and obtain more credible estimates, we adopt a Bartik-style instrumental variables (IV) approach to construct exogenous measures of regional export shocks. Based on this empirical strategy, we estimate the association between trade exposure and child morbidity outcomes. Furthermore, we explore the potential mechanisms underlying this relationship from four perspectives: parental income, parental employment rate, parental work intensity, and the prevalence of left-behind children. By focusing on the unintended health consequences of trade liberalization, particularly for children in developing countries, this paper contributes to the emerging literature at the intersection of globalization, labor dynamics, and public health.

The remainder of the paper is organized as follows. Section 2 reviews the relevant literature from international trade, health economics, and labor economics, and outlines the conceptual mechanisms linking trade to child health. Section 3 introduces the data sources and details the construction of key variables. Section 4 presents the empirical strategy, including the development of the Bartik-style export shock instrument—which effectively addresses endogeneity concerns—and a summary of the descriptive statistics. Section 5 reports the core empirical results, including the baseline Probit regressions, robustness checks based on alternative definitions of child age, and subgroup heterogeneity analyses. Section 6 investigates the mediating channels through which export shocks may affect child health. Section 7 concludes by summarizing the key findings, discussing policy implications, and reflecting on the study’s limitations and directions for future research.

## 2 Literature review

Given the relative scarcity of research in this field (12, 13) and the lack of consensus in existing studies (14, 15), this paper systematically reviews the literature on trade and child health. Early research on this topic primarily focused on macro-level health indicators. For example, studies using cross-sectional data from 100 to 130 countries have empirically examined the impact of trade liberalization on child health (12). Their findings suggest that trade does not harm child health; rather, it benefits child health by accelerating GDP growth. A 15-percentage-point increase in the trade-to-GDP ratio was associated with a reduction of approximately four infant deaths per 1,000 live births and a decrease of four deaths per 1,000 children under the age of five. Similar conclusions have been drawn in studies conducted in other countries (14–16). Conversely, Qadir and Majeed (17) used data from Pakistan spanning 1975–2016 to investigate the impact of trade liberalization on health indicators. Their findings indicate that a 1% increase in trade openness significantly reduces life expectancy by 0.05 years and increases infant mortality by 0.47 per 1,000 live births. They argue that, in the context of Pakistan, trade liberalization has a detrimental effect on health capital (17).

In recent years, researchers have also begun exploring the environmental pollution channels through which trade affects health. For instance, Bombardini and Li (18) utilized data from Chinese prefecture-level cities to examine the impact of exports on infant mortality. By constructing variables for export shocks and export-induced environmental pollution shocks, they found that while export shocks contributed to lower infant mortality, the results were not robust; however, export-induced pollution shocks significantly increased infant mortality. Notably, these studies all use infant mortality rates as a proxy for child health. However, infant health is highly volatile, and mortality rates may be influenced by random factors, leading to measurement bias in assessing regional child health levels. As trade liberalization has deepened, scholars have increasingly adopted a micro-level perspective to examine the relationship between trade and health. Colantone et al. (19, 20) used data from the British Household Panel Survey to empirically analyze the impact of import competition on workers’ mental health. Their results show that trade exposure increases psychological distress among workers, potentially due to deteriorating labor market conditions, higher unemployment risk, and lower wage growth, all of which contribute to poorer mental health outcomes. Similarly, Hummels et al. (21) explored the effects of exogenous export growth on workers and found that increased exports led to longer working hours, fewer sick leave days, and higher injury rates, particularly among female workers. Studies using China as a sample have reached similar conclusions (22, 23).

Clearly, research directly examining the impact of trade liberalization on child health in China remains limited. This study seeks to bridge this gap by adopting an interdisciplinary perspective to explore the relationship and underlying mechanisms between trade and child health, drawing insights from various intersecting fields.

Firstly, from the perspective of international trade and labor economics, export growth generates employment opportunities, increasing parental income levels, which in turn influences child health (24). However, employment-driven rural-to-urban migration in China has led to a rise in the number of “left-behind children,” who experience prolonged parental absence. Studies indicate that parental migration negatively affects the health of left-behind children, with the absence of mothers being particularly detrimental (25–29). Similar findings have been reported in global research. For example, Fellmeth et al. (30) investigated the impact of parental migration on left-behind children in low-and middle-income countries (LMICs), revealing that these children face an elevated risk of depression compared to their non-migrant counterparts. A review of the literature suggests that trade influences child health capital through multiple channels, including parental income, work intensity, and employment status. Therefore, we hypothesize that export expansion may improve child health by increasing parental income while simultaneously exerting negative effects through the creation of left-behind children and reduced parental caregiving time due to increased work intensity.

Secondly, research at the intersection of international trade and health economics suggests that trade affects children’s health by influencing food prices, which in turn impacts their nutritional intake (31). Furthermore, as export expansion generates higher government tax revenues, public services such as healthcare and education improve. According to Grossman’s (32, 33) health demand model, higher educational attainment enhances the efficiency of health production as human capital, thereby reducing the shadow price of health and increasing both the flow and stock of health capital, ultimately leading to better health outcomes.

Thirdly, the interactions between trade and environmental pollution are considered. The relationship between trade openness and the environment is debated within three competing perspectives: trade openness benefits the environment, trade openness harms the environment, and the relationship is complex and uncertain (34–38). Cropper (39) incorporated air pollution as a key factor influencing health into Grossman’s theoretical model, thereby establishing an analytical framework for studying the health effects of air pollution (39). Subsequent studies have reached a broad consensus that environmental pollution negatively impacts health outcomes (40–44).

**Figure 3:**
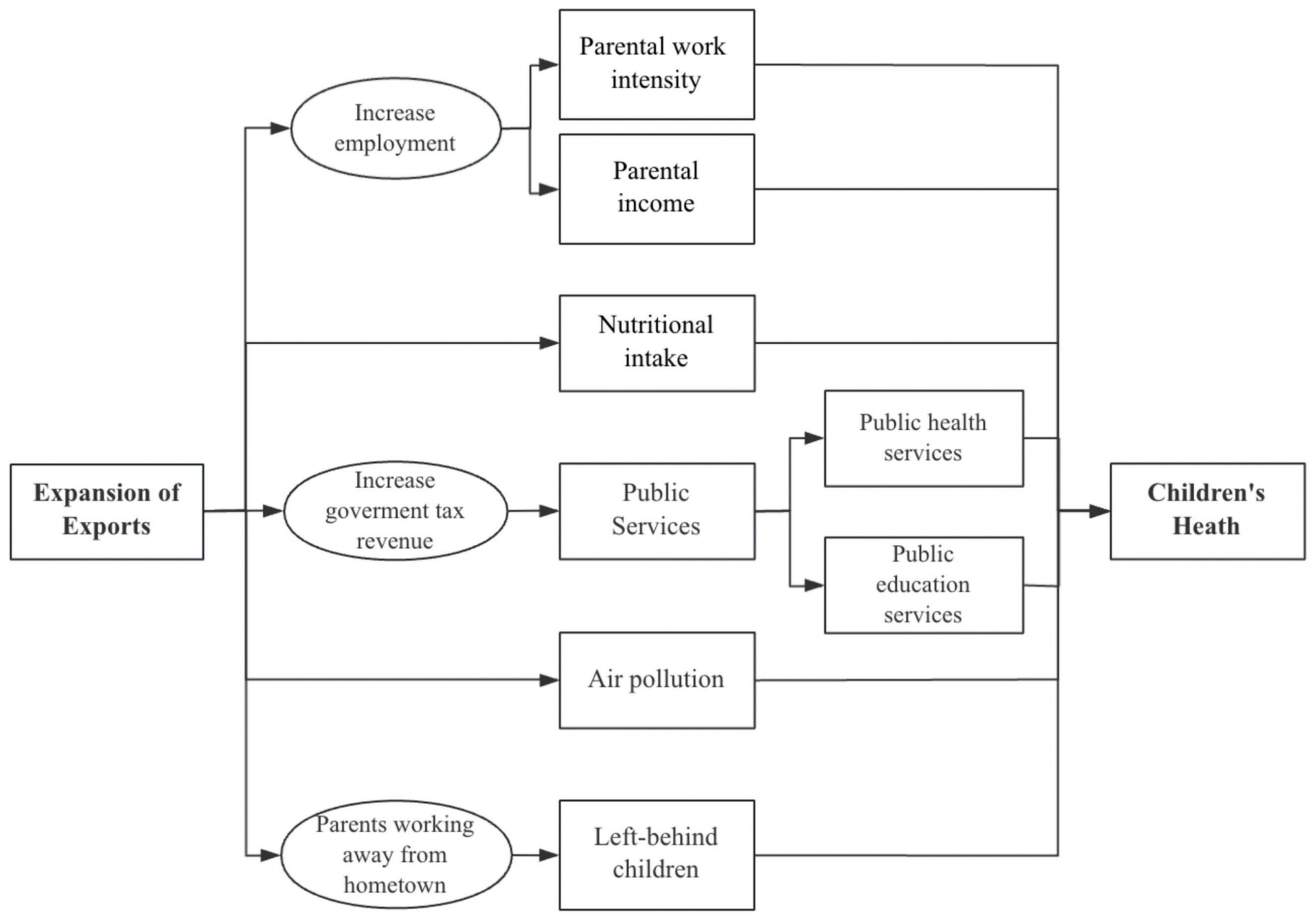
Pathways of export expansion on child health. Source: compiled by the authors.

In summary, export expansion can influence child health capital through multiple indirect channels, including government tax revenues, child nutrition, environmental quality, parental income, work intensity, employment status, and the emergence of left-behind children (see Figure 3). In recent years, research examining the relationship between exports and health capital has begun to emerge. For example, Zhang et al. (23) investigate the relationship between environmental pollution, trade openness, and the health of middle-aged and older adults individuals in China, using panel data from 111 prefecture-level cities. Their study identifies a significant adverse effect of environmental pollution on health, with evidence of regional heterogeneity and a single-threshold nonlinear relationship. Moreover, trade openness modifies this relationship, initially weakening and subsequently reinforcing the negative health impact of pollution. These findings highlight the need for differentiated environmental and trade policies that account for local pollution levels, degrees of openness, and regional characteristics (45). Bombardini and Li (18) provided empirical evidence that export-induced air pollution significantly increases infant mortality, whereas export shocks alone contribute to lower infant mortality, though the results are not robust. Other studies have also explored the relationship between trade and adult health (46, 47).

Although these studies offer valuable insights, the relationship between trade and child health, as well as the underlying mechanisms, remains underexplored. To address this research gap, this paper employs data from the China Health and Nutrition Survey (CHNS) and constructs an export shock variable using the Bartik-IV approach to empirically examine the impact of export expansion on child health. Furthermore, we investigate the potential mechanisms through which trade affects child health, specifically focusing on parental regular income, employment rate, work intensity, and the presence of left-behind children. This study contributes to the emerging literature on trade liberalization and child health capital by providing empirical evidence from a developing country context.

## 3 Data and variables

### 3.1 Dataset

This study primarily utilizes three categories of data: trade data, micro-level individual data, and national and regional employment data.

The key micro-level data source is the China Health and Nutrition Survey (CHNS), a publicly available longitudinal dataset jointly conducted by the University of North Carolina at Chapel Hill and the Chinese Center for Disease Control and Prevention. The CHNS was originally designed to evaluate the effectiveness of health, nutrition, and family planning policies across diverse socio-economic regions in China. Over time, it has evolved into one of the most authoritative and widely used databases for examining health and development issues in China. The CHNS adopts a multistage, stratified, and cluster random sampling method across a range of provinces that differ substantially in geography, economic development, public infrastructure, and health profiles. In the first stage, nine provinces were selected to represent China’s eastern, central, and western regions. Within each province, four counties (one high-income, two middle-income, and one low-income) were randomly selected to form the rural sample, while the provincial capital and a lower-income city were chosen to form the urban sample. In the subsequent stages, approximately 220 communities were sampled across these regions, and about 20 households per community were randomly selected. The final sample includes approximately 4,400 households and over 19,000 individuals. The survey was started in 1989, and several rounds of surveys were conducted in 1991, 1993, 1997, 2000, 2004, 2006, 2009, 2011, and 2015, and this paper chooses the data of 2004, 2006, 2011 and 2015 for empirical investigation.

Importantly, CHNS collects a rich array of variables related to individual and household characteristics, including health status, anthropometric indicators, access to medical services, insurance coverage, labor market status, income, and consumption behavior. It also includes retrospective questions on short-term health episodes such as child morbidity, which are crucial for our empirical identification strategy. Although CHNS does not aim for full national representativeness, its stratified sampling design ensures substantial heterogeneity across provinces and socioeconomic strata. As such, the dataset has been extensively used in empirical studies published in leading journals to evaluate public health policies, income-health gradients, and nutrition dynamics. Given the breadth of its coverage and its longitudinal structure, CHNS provides a robust basis for examining the association between trade shocks and child health outcomes across different population subgroups in China.^3^

Secondly, trade data for this study are obtained from the United Nations Commodity Trade Database (UN Comtrade Database).^4^ To align with the micro-level health data, we calculate export fluctuations by measuring the difference in exports between two consecutive years.^5^ We also match the HS 2-digit industry codes from the UN Comtrade Database with the major industry classification codes from China’s National Economic Industry Classification Standard (GB/T 4754) to construct industry-specific export shock data for each year.

Finally, employment data for different industries in the Chinese manufacturing sector are sourced from the 2000 China 0.95‰ Sample Census Microdata.

In summary, we utilize data from the CHNS and UN Comtrade Database to construct an unbalanced panel dataset spanning four periods. It is important to note that this study follows the definition of “children” as stated in Article 1 of the United Nations Convention on the Rights of the Child, referring to individuals aged 18 years or younger.

### 3.2 Variables of export shock

First, following the methodology of Bartik (48) and Autor et al. (49) for constructing regional import shocks, this study constructs a regional export shock variable. Following standard practice in the literature, we use total regional employment as a proxy for total regional output and employment by industry as a proxy for industry-specific output. Specifically, we calculate the share of employment in a given industry within a province relative to the national employment in that industry and use this as a proxy for the industry’s output share. This proxy is then combined with national-level export data to construct a measure of per capita export change at the regional level, which serves as our export shock variable. Specifically, we first estimate the impact of export-induced demand shocks on the total output of region *i* in period *t*, calculated as follows:


(1) $$\sum_{d}\frac{\psi_{\mathrm{id}}}{\psi_{d}}\frac{\Delta \varphi_{\mathrm{dt}}}{O_{i}}$$

As shown in Equation 1, $O_{i}$ represents the total output of region *i*, $\Delta \varphi_{\mathrm{dt}}$ denotes the export growth of sector *d* in period *t*, $\psi_{\mathrm{id}}$ refers to the sales of sector *d* in region *i*, and $\psi_{d}$ represents the total national sales of sector *d*. It is evident that the higher the relative export growth of a given sector compared to total output, the larger the proportion of that sector’s sales in the national total, and consequently, the greater the exposure to export shocks.

A practical challenge arises due to the unavailability of comprehensive data on sectoral total output and total sales across regions. To address this, we adopt total regional employment as a proxy for regional total output and sectoral employment as a proxy for sectoral output. Based on this approach, we ultimately derive the per capita export variation, which serves as the regional export shock variable:


(2) $$\text{Export}\_\text{Shoc}k_{\mathrm{it}}=\sum_{d}\;\frac{E_{\mathrm{idt}}}{E_{\mathrm{dt}}}\frac{\Delta \varphi_{\mathrm{dt}}}{E_{\mathrm{it}}}$$

In Equation 2, $\text{Export}\_\text{Shoc}k_{\mathrm{it}}$ represents the regional export shock variable constructed in this study. $\Delta \varphi_{\mathrm{dt}}$ denotes the export variation of sector *d* in period *t*, $E_{\mathrm{it}}$ is the total employment in region *i* during period *t*, and $\frac{E_{\mathrm{idt}}}{E_{\mathrm{dt}}}$ represents the share of sector *d*’s employment in region *i* relative to the national employment of sector *d* in period *t*. The construction of $\text{Export}\_\text{Shoc}k_{\mathrm{it}}$ follows the widely adopted “Bartik instrument,” which effectively mitigates endogeneity concerns in empirical analysis (31, 48), thus providing a robust foundation for further mechanism analysis (49, 50). Moreover, to ensure exogeneity and stability, this study primarily employs the employment structure from the year 2000 to construct the regional export shock variable, as specified in Equation 3:


(3) $$\text{Exportshoc}k_{\mathrm{it}}=\sum_{d}\;\frac{E_{\mathrm{id},2000}}{E_{d,2000}}\frac{\Delta \varphi_{\mathrm{dt}}}{E_{i,2000}}$$

$\frac{E_{\mathrm{id},2000}}{E_{d,2000}}$ represents the proportion of employment in sector *d* within region *i* relative to the total national employment in sector *d* in the year 2000. $\frac{\Delta \varphi_{\mathrm{dt}}}{E_{i,2000}}$ denotes the portion of export variation in sector *d* that is allocated to region *i*, which is then normalized by the total population of region *i* in the year 2000.

## 4 Empirical strategy and description statistic

### 4.1 Model

To ensure robustness, we construct an unbalanced panel dataset at the individual level and estimate the model as follows:


(4) $$\begin{array}{l} \text{Childhealt}h_{\mathrm{idt}}=a+\text{βExport}\_\text{Shoc}k_{\mathrm{idt}}+\lambda_{1}Z_{\mathrm{idt}}^{\text{individual}}\\ +\lambda_{2}Z_{\mathrm{idt}}^{\text{parental}}+\lambda_{3}Z_{\mathrm{idt}}^{\text{household}}+\gamma_{c}+\gamma_{t}+\varepsilon_{\mathrm{idt}} \end{array}$$

In Equation 4, $\text{Export}\_\text{Shoc}k_{\mathrm{idt}}$ represents the export shock experienced by individual *i* in region *d* during period *t*. The coefficient *β* captures the impact of trade liberalization-induced export shocks on local children’s health outcomes. Specifically, $\text{Childhealt}h_{\mathrm{idt}}$ is the health status of child *i* in region *d* at time *t* is measured by morbidity, as derived from survey data.^6^

The control variables include: individual characteristics $Z_{\mathrm{idt}}^{\text{individual}}$ , for example, age, gender, household registration status, years of education, height, weight, health insurance coverage, and whether the child is an only child. Parental characteristics $Z_{\mathrm{idt}}^{\text{parental}}$ , for example, father’s years of education, height, weight, and health insurance coverage; mother’s years of education, height, weight, and health insurance coverage. Household characteristics $Z_{\mathrm{idt}}^{\text{household}}$ , for example, household size, access to indoor tap water, and availability of an indoor flush toilet, lighting and indoor clear energy. Where: $\gamma_{c}$ represents individual fixed effects, controlling for time-invariant individual characteristics; $\gamma_{t}$ represents year fixed effects, capturing common temporal trends across regions; $\varepsilon_{\mathrm{idt}}$ is the random error term.^7^

### 4.2 Description statistic

Following the definition in Equation 4, we restrict the sample to children aged 18 years or younger and present the summary statistics of the key variables in Table 1. The descriptive results indicate that the average child morbidity rate in the sample is approximately 7%, with the mean age of children being around 12 years. Girls account for 47% of the sample, and urban children represent 35%. In terms of access to healthcare, 45% of children have health insurance coverage, while 55% are uninsured. Additionally, about 13% of the children are the only child in their household. The average height and weight of children are 146.99 cm and 40.51 kg, respectively.

**Table 1:** Description of variables and descriptive statistics.

Variables		Obs	Mean (Std.)	Variable description
Independent variable	Export shock	5,234	5.02 (0.94)	Continuous variable
Dependent variable	Child morbidity (%)	9,163	0.07 (0.25)	Sickness or injury in the past 4 weeks = 1, otherwise = 0
Individual variable	Age	9,226	12.16 (3.56)	Continuous variable
	Female (%)	9,226	0.47 (0.50)	Female = 1; male = 0
	City (%)	9,109	0.35 (0.48)	Urban = 1; rural = 0
	Region	9,226	2.00 (0.76)	Discrete variable^1^
	Medical insurance (%)	9,222	0.45 (0.49)	With medical insurance = 1; otherwise = 0
	Education	8,043	2.03 (0.81)	Discrete variable^2^
	Only child (%)	9,226	0.13 (0.33)	Only child = 1; otherwise = 0
	Height (cm)	8,408	146.99 (18.39)	Continuous variable
	Weight (kg)	8,430	40.51 (14.60)	Continuous variable
Household variable	Tap water (%)	9,124	0.73 (0.45)	Household has tap water = 1; otherwise = 0
	Lighting (%)	9,113	0.99 (0.10)	Household has electric lighting = 1; otherwise = 0
	Indoor clear energy (%)	9,101	0.53 (0.50)	Household has indoor clean energy = 1; otherwise = 0
	Flush toilet (%)	9,115	0.43 (0.50)	Household has flush toilet = 1; otherwise = 0
Parental variable	Educated years of father (years)	8,477	9.24 (3.61)	Discrete variable
	Height of father (cm)	8,059	167.05 (6.47)	Continuous variable
	Weight of father (kg)	8,008	65.85 (11.45)	Continuous variable
	Medical insurance of father (%)	8,676	0.64 (0.47)	With medical insurance = 1; otherwise = 0
	Education of mother (years)	8,611	7.99 (4.26)	Discrete variable
	Height of mother (cm)	8,682	156.54 (6.09)	Continuous variable
	Weight of mother (kg)	8,652	57.37 (9.45)	Continuous variable
	Medical insurance of mother (%)	9,041	0.64 (0.48)	With medical insurance = 1; otherwise = 0
Other variables	Regular wage of father (%)	5,226	0.61 (0.50)	Father has a regular wage income = 1; otherwise = 0
	Regular wage of mother (%)	4,239	0.57 (0.48)	Mother has a regular wage income = 1; otherwise = 0
	Work intensity of father (hours)	5,779	33.65 (33.86)	Continuous variable Average hours worked per week
	Work intensity of mother (hours)	4,479	30.39 (33.41)	Continuous variable Average hours worked per week
	Work of father (%)	8,670	0.75 (0.43)	Presently father has a job = 1; otherwise = 0
	Work of mother (%)	9,041	0.60 (0.50)	Presently father has a job = 1; otherwise = 0
	Left child (%)	9,226	0.10 (0.31)	Left-behind children = 1; otherwise = 0

“()” represents standard deviation. Source: compiled by the author according to China Health and Nutrition Survey. ^1^Region: in this paper, region is set as a categorical variable, where East = 1, Center = 2; West = 3, and “East” is the control group. The basis of division refers to the division standard of the China Bureau of Statistics (https://www.stats.gov.cn/zt_18555/zthd/sjtjr/dejtjkfr/tjkp/202302/t20230216_1909741.htm Date of application: 15.10.2024). ^2^Education: in this paper, education of children is set as a categorical variable, where Illiterate = 1, Primary school = 2; Middle school, secondary school and above = 3, and “Illiterate “is the control group.

In terms of household level characteristics, 43% of children live in homes equipped with an indoor flush toilet, and 73% have access to piped water. Almost all households (99%) have electricity, and 53% use clean indoor energy sources such as gas or electricity for cooking.

We also report descriptive statistics for variables related to the mechanism analysis. The results show that 61% of fathers and 57% of mothers in the sample have regular wage employment. Regarding broader employment participation, 75% of fathers and 60% of mothers are employed. The average number of working hours per week is 33.65 for fathers and 30.39 for mothers. Furthermore, 10% of the children in the sample are categorized as left-behind, meaning they are not currently living with one or both parents due to parental labor migration.

Figure 2 provides an overview of the temporal trend in child morbidity rates across subgroups defined by gender and hukou (household registration) status over the period 1991–2015. The data show a general upward trend in reported morbidity, with girls experiencing a more pronounced increase compared to boys. Similarly, both urban and rural children saw rising morbidity rates, but the increase was notably steeper among rural children.

These descriptive patterns provide an overview of the sample characteristics and key health-related indicators. While they do not imply any causal relationships, they offer important contextual information that helps to frame the subsequent empirical analysis. In the next section, we proceed to estimate the association between export shocks and child morbidity using probit models and a Bartik-IV identification strategy.

## 5 Results

### 5.1 Basic regression results

To estimate the impact of export shocks on child health outcomes, we adopt a stepwise modeling approach designed to assess both the stability and robustness of our estimates. We begin by estimating a series of probit models without fixed effects. In these baseline specifications, individual-, household-, and parental-level control variables are sequentially added in columns (1) through (3). This progression allows us to observe how the estimated relationship between export shocks and child morbidity evolves as additional layers of potential confounders are introduced. These models help establish the initial association and offer a benchmark for subsequent robustness checks.

Recognizing that unobserved heterogeneity may persist—such as time-invariant differences in local infrastructure, healthcare access, or socioeconomic development—we further extend our analysis by including both community and year fixed effects in columns (4) through (6). This specification controls for region-specific characteristics that do not change over time, as well as macro-level temporal shocks that might simultaneously affect all regions. By comparing estimates across models with and without fixed effects, we aim to disentangle the effect of export shocks from potentially confounding contextual variables. This modeling strategy aligns with standard empirical practices in applied microeconomics and enhances the credibility of causal inference (31).

The estimation results are presented in Table 2. In the baseline models without fixed effects, we find weakly significant positive associations between export shocks and child health, but the results are sensitive to the inclusion of control variables. Once community and year fixed effects are incorporated, the direction of the relationship reverses and becomes consistently negative. Specifically, in the fully specified model (Probit 6), which controls for individual-, household-, and parental-level characteristics as well as fixed effects, a one-standard-deviation increase in export shocks is associated with a 14% reduction in the probability of child morbidity, significant at the 10% level.

**Table 2:** The impact of export shocks on the health of children.^1^

Variables	Morbidity of children					
	Probit (1)	Probit (2)	Probit (3)	Probit (4)	Probit (5)	Probit (6)
Export shock	0.07* (0.05)	0.06 (0.05)	0.09 (0.05)	−0.12* (0.06)	−0.13** (0.06)	−0.14* (0.07)
Individual Controls	Y	Y	Y	Y	Y	Y
Household Controls	N	Y	Y	N	Y	Y
Parental Controls	N	N	Y	N	N	Y
Community FE	N	N	N	Y	Y	Y
Year FE	N	N	N	Y	Y	Y
Observations	3,703	3,635	2,956	3,643	3,576	2,867
R-squared	0.02	0.02	0.02	0.03	0.03	0.04

All regressions include a constant term. Robust standard errors are reported in parentheses. Probit (1–3) sequentially incorporate individual-, household-, and parental-level control variables without fixed effects. Probit (4–6) additionally control for community and year fixed effects. Statistical significance is denoted by *p < 0.10, **p < 0.05, and ***p < 0.01. ^1^Data notes: due to space constraints, the estimation results of the control variables are not reported in this paper and are kept for reference.

This reversal underscores the importance of accounting for unobserved heterogeneity: failing to control for region-specific or time-specific confounders may lead to biased or misleading estimates. The final specification suggests a robust and negative association between trade exposure and child morbidity, implying that increased exports may improve child health outcomes, potentially through labor market or income-related mechanisms. We explore these channels in greater depth in Section 6.

### 5.2 Robustness check

To assess the robustness of our baseline findings, we conduct a set of sensitivity analyses. In the main specification, the sample includes children aged 18 years or younger, consistent with Article 1 of the United Nations Convention on the Rights of the Child, which defines a child as “every human being below the age of 18 years.” Accordingly, we treat individuals under 18 as children in our primary analysis.

However, the definition of childhood varies across empirical studies, with some adopting lower age thresholds such as 15 or 14 years old. To ensure that our results are not sensitive to the choice of age cutoff, we re-estimate the model using two alternative definitions: children aged 15 years and under, and children aged 14 years and under. These specifications are commonly adopted in the public health and development literature, particularly in studies using CHNS or similar demographic data.

The estimation results under both alternative definitions remain qualitatively consistent with those of the baseline model. In all cases, export shocks are significantly associated with reduced child morbidity, and the signs and magnitudes of the estimated coefficients do not materially change. This reinforces the conclusion that our findings are not driven by a particular age specification.

Overall, the robustness checks support the internal consistency of our results and suggest that the observed negative association between export shocks and child morbidity is not sensitive to alternative modeling choices or sample definitions.

### 5.3 Heterogeneity analysis

To further explore the distributional effects of export shocks on child health, we conduct heterogeneity analyses across three key dimensions: gender, household registration (urban and rural), and geographic region (eastern, central, and western China). The results are presented in Table 3.

**Table 3:** Heterogeneous effects of export shocks on child health.

Variables	Gender		Household registration		Region		
	Male	Female	Urban	Rural	East	Middle	West
Export shock	−0.23** (0.10)	−0.01 (0.10)	−0.26** (0.11)	−0.06 (0.11)	0.09 (0.13)	−0.11 (0.12)	−0.56*** (0.18)
Individual Controls	Y	Y	Y	Y	Y	Y	Y
Household Controls	Y	Y	Y	Y	Y	Y	Y
Parental Controls	Y	Y	Y	Y	Y	Y	Y
Community FE	Y	Y	Y	Y	Y	Y	Y
Year FE	Y	Y	Y	Y	Y	Y	Y
Observations	1,553	1,343	1,053	1854	819	1,156	919
R-squared	0.06	0.05	0.07	0.05	0.05	0.08	0.12

All regressions include a constant term. Robust standard errors are reported in parentheses. All specifications control for individual-, household-, and parental-level covariates, as well as community and year fixed effects. Columns (1–7) report results from heterogeneity analyses by gender (male, female), household registration (urban, rural), and region (east, middle, west). Statistical significance is denoted by *p < 0.10, **p < 0.05, and ***p < 0.01.

The gender-based subsample regressions reveal that export shocks significantly reduce child morbidity among boys (coefficient = −0.23, *p* < 0.05), while the effect is statistically insignificant for girls. This gender gap may reflect entrenched son preference and intra-household disparities in health-related investments. Prior studies have shown that in many developing countries, including China, son preference manifests in unequal allocation of household resources such as nutrition, healthcare, and education, often to the disadvantage of girls (51, 52). Hafeez and Quintana-Domeque (53) further report adverse impacts on female child height and health outcomes under son preference regimes. In China, this pattern is compounded by institutional and social factors. For instance, Sun et al. (54) demonstrate that rural families are more likely to bring school-aged sons, rather than daughters, to urban areas for education, given limitations in access to public services under the hukou system. This unequal migration decision implies broader disparities in parental attention and investment in children’s human capital, including health.

In terms of household registration, export shocks are significantly associated with reduced child morbidity in urban households (coefficient = −0.26, *p* < 0.05), but not in rural ones. This divergence is likely attributable to substantial disparities in access to healthcare and public health services. Urban areas benefit from higher densities of medical personnel, better-equipped hospitals, and more comprehensive public insurance systems. Wang et al. (55) find that improvements in urban healthcare resources significantly influence medical service utilization, while similar expansions in rural areas have more limited effects. Yan and Zhang (56) emphasize that China’s rural health system remains underfunded and poorly equipped, making it harder for rural households to translate economic gains from trade into tangible health improvements. Moreover, institutional constraints such as the hukou system restrict rural children’s access to urban health services, further deepening urban–rural inequalities.

Regional heterogeneity also emerges export shocks significantly reduce child morbidity in western China (coefficient = −0.56, *p* < 0.01), while no statistically significant effects are observed in the eastern and central regions. This pattern likely reflects regional differences in industrial structure and trade dependence. Western provinces, often less economically developed, are more reliant on labor-intensive export sectors. Thus, trade liberalization may generate stronger local employment and income gains, with more pronounced downstream benefits for household welfare and child health. Additionally, the marginal utility of income may be higher in poorer regions, enhancing the health effects of trade-induced income shocks. This interpretation is consistent with the broader literature on regional inequality in China (57).

**Figure 4:**
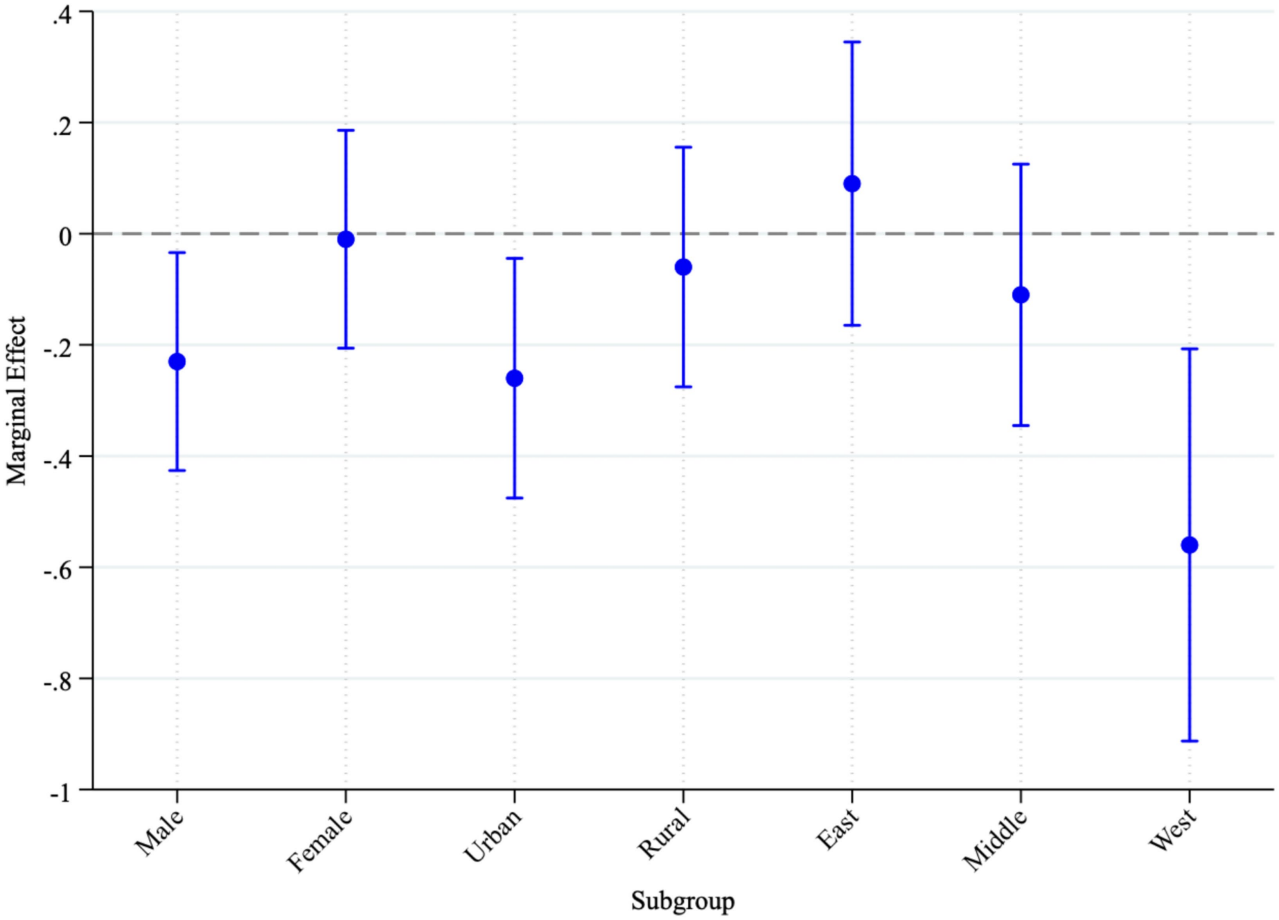
Marginal effects of export shocks on child morbidity by subgroup. Source: compiled by the authors.

In summary, the heterogeneity analysis suggests that the health benefits of export expansion are not evenly distributed. Boys, urban children, and those living in western China appear to benefit more, highlighting the importance of demographic, institutional, and geographic contexts in shaping the link between trade and child health. To further illustrate the subgroup differences observed in the heterogeneity analysis, Figure 4 presents the estimated marginal effects of export shocks on child morbidity by gender, household registration, and region.

This plot translates the probit regression coefficients into interpretable marginal effects, offering a clearer understanding of the magnitude and direction of impact. The results show that export shocks significantly associated with lower child morbidity among boys, urban children, and those residing in western China, with statistically significant negative marginal effects and narrow confidence intervals. In contrast, the effects for girls, rural children, and children in eastern or central regions are not statistically significant.

## 6 Mechanism analysis

As discussed in the introduction, export shocks may influence children’s health through multiple channels, including taxation, environmental conditions, household economic status, and the left-behind children phenomenon. Based on data availability and theoretical relevance, this study focuses on four primary mediating factors: parents’ regular wage income, parental employment rate, parental work intensity, and whether the child is left behind due to parental migration. To formally test these mechanisms, we adopt a structured mediation analysis framework, following standard econometric practice (58, 59). The analysis proceeds in three steps:

*Step 1*: estimate the total effect of export shocks on child morbidity by regressing the dependent variable on the independent variable, controlling for relevant covariates. This establishes whether a statistically significant baseline association exists.

*Step 2*: regress each potential mediator (e.g., wage income, employment status, work intensity, left-behind status) on the export shock variable to examine whether the independent variable affects the mediator.

*Step 3*: include both the export shock variable and the mediator in a single regression to estimate the indirect effect. A reduction in the export shock coefficient and a statistically significant mediator coefficient provides evidence of a partial mediation effect.

The corresponding empirical results are reported in Table 4 through 7, each addressing a distinct mediating mechanism.

**Table 4:** Mediation effects: regular wage of parents.

Variables	Regular wage of father			Regular wage of mother		
	Probit (1)	Probit (2)	Probit (3)	Probit (4)	Probit (5)	Probit (6)
Export shock	−0.14* (0.07)	0.09* (0.13)	−0.18* (0.09)	−0.14* (0.07)	0.22** (0.09)	−0.22** (0.11)
Individual controls	Y	Y	Y	Y	Y	Y
Household controls	Y	Y	Y	Y	Y	Y
Parental controls	Y	Y	Y	Y	Y	Y
Community FE	Y	Y	Y	Y	Y	Y
Year FE	Y	Y	Y	Y	Y	Y
Observations	2,867	1842	1830	2,867	1,471	1,424
R-squared	0.04	0.27	0.07	0.04	0.37	0.09

All regressions include a constant term. Robust standard errors are reported in parentheses. All specifications control for individual-, household-, and parental-level covariates, as well as community and year fixed effects. Probit (1–3) and (4–6) correspond to the three-step mediation models assessing the roles of the father’s and mother’s regular wages, respectively. Statistical significance is denoted by *p < 0.10, **p < 0.05, and ***p < 0.01.

### 6.1 Regular wage of parents

To begin the mechanism analysis, we examine whether the regular wage income of parents serves as a mediating factor in the relationship between export shocks and child morbidity. Table 4 presents the results of the three-step mediation model, separately for fathers and mothers.

In Step 2, Probit (2) and Probit (5) indicate that exposure to export shocks is positively and significantly associated with an increase in parental regular wage income. The effect is statistically significant at the 10% level for fathers and at the 5% level for mothers. This finding suggests that trade liberalization may enhance household income by improving labor market conditions and increasing wage opportunities, particularly for female workers.

In Step 3, the estimates from Probit (3) and Probit (6) show that export shocks are associated with reductions in the likelihood of child morbidity by approximately 4% points through increased regular wage income of fathers, and by about 8% points through increased regular wage income of mothers. Both effects are statistically significant at the 10 and 5% levels, respectively. The stronger mediating effect observed through maternal earnings may reflect the higher marginal impact of mothers’ income on child health investment. This result is consistent with the literature on intra-household resource allocation, which suggests that maternal income is more directly linked to child wellbeing.

Overall, the findings indicate that improvements in household income, particularly maternal income, constitute an important channel through which export shocks contribute to better child health outcomes. The observed gender difference in mediation strength underscores the importance of accounting for intra-household dynamics when evaluating the health implications of economic globalization.

### 6.2 Employment rate of parents

The three-step mediation analysis in Table 5 shows that export shocks improve child health by boosting both paternal and maternal employment, but with different magnitudes.

**Table 5:** Mediation effects: employment rate of parents.

Variables	Paternal employment			Maternal employment		
	Probit (1)	Probit (2)	Probit (3)	Probit (4)	Probit (5)	Probit (6)
Export shock	−0.14* (0.07)	0.12** (0.53)	−0.14* (0.07)	−0.14* (0.07)	0.06* (0.05)	−0.13* (0.07)
Individual controls	Y	Y	Y	Y	Y	Y
Household controls	Y	Y	Y	Y	Y	Y
Parental controls	Y	Y	Y	Y	Y	Y
Community FE	Y	Y	Y	Y	Y	Y
Year FE	Y	Y	Y	Y	Y	Y
Observations	2,867	2,967	2,905	2,867	2,973	2,911
R-squared	0.04	0.14	0.04	0.04	0.09	0.04

All regressions include a constant term. Robust standard errors are reported in parentheses. All specifications control for individual-, household-, and parental-level covariates, as well as community and year fixed effects. Probit (1) through Probit (3) correspond to the three-step mediation model examining the role of paternal employment, while Probit (4) through Probit (6) refer to the mediation pathway through maternal employment. Statistical significance is denoted by *p < 0.10, **p < 0.05, and ***p < 0.01.

As shown in Table 5, the second stage of the mediation analysis, represented by Probit (2) and Probit (5), suggests that export shocks increase paternal employment by 12% points, with the effect statistically significant at the 5% level, and increase maternal employment by 6% points, significant at the 10% level. In the third step (Probit 3 and Probit 6), these employment gains are associated with reductions in child morbidity of 14 and 13% points through the paternal and maternal channels, respectively, both significant at the 10% level.

While both pathways yield health benefits, the stronger mediation via paternal employment likely reflects that fathers’ additional income can be allocated to health-related goods and services without competing demands on caregiving time. Employed mothers, by contrast, often balance paid work with household responsibilities, which may limit the time available for direct child care and thus slightly attenuate the positive spillover from their earnings. These findings are consistent with evidence showing that the health effects of parental employment hinge not only on income gains but also on how parents allocate time to childcare and household duties (60, 61).

### 6.3 Work intensity of parents

This section investigates whether the intensity of parental labor serves as a mediating channel in the relationship between export shocks and child morbidity. As shown in Table 6, the second step of the mediation model, represented by Probit (2) and Probit (5), indicates that export shocks are positively associated with increased work intensity for both fathers and mothers, with statistical significance at the 5 and 10% levels, respectively.

**Table 6:** Mediation effects: work intensity of parents.

Variables	Work intensity of father			Work intensity of mother		
	Probit (1)	Probit (2)	Probit (3)	Probit (4)	Probit (5)	Probit (6)
Export shock	−0.14* (0.07)	0.14** (0.06)	−0.14* (0.08)	−0.14* (0.07)	0.08* (0.05)	−0.13 (0.08)
Individual controls	Y	Y	Y	Y	Y	Y
Household controls	Y	Y	Y	Y	Y	Y
Parental controls	Y	Y	Y	Y	Y	Y
Community FE	Y	Y	Y	Y	Y	Y
Year FE	Y	Y	Y	Y	Y	Y
Observations	2,867	2,606	2,535	2,867	2,603	2,526
R-squared	0.04	0.15	0.05	0.04	0.10	0.06

All regressions include a constant term. Robust standard errors are reported in parentheses. All specifications control for individual-, household-, and parental-level covariates, as well as community and year fixed effects. Probit (1) through (3) correspond to the three-step mediation model for paternal work intensity, while Probit (4) through (6) represent the mediation pathway for maternal work intensity. Statistical significance is denoted by *p < 0.10, **p < 0.05, and ***p < 0.01.

In the third step, the estimated coefficients of Probit (3) and Probit (6) suggest divergent patterns between paternal and maternal labor intensity. Specifically, the results indicate that greater paternal work intensity significantly reduces the probability of child morbidity, suggesting a positive mediation pathway. However, the coefficient for maternal work intensity becomes statistically insignificant, indicating that increased maternal work intensity no longer significantly reduces child morbidity.

These gender-differentiated effects may reflect the dual burden often experienced by working mothers, who must balance paid labor with unpaid caregiving responsibilities. As maternal work intensity increases, the time and attention available for child care may decrease, thereby attenuating or offsetting the potential health benefits derived from higher household labor force participation. This interpretation aligns with broader literature highlighting that the health effects of parental employment depend not only on income but also on intra-household time allocation and caregiving dynamics (61–63). Empirical evidence from China also reinforces this view. Using data from the China Health and Nutrition Survey (CHNS), Gu and Liu (63) find that maternal employment during a child’s early developmental window (age 0–3) is significantly associated with poorer health outcomes, due to reduced maternal caregiving time during this critical period (64). Similarly, Ding (65) reports that among children under age two, those whose mothers work more than 40 h per week face a 55% higher risk of overweight and obesity compared to children of non-working mothers—especially in urban areas and among more educated mothers. These findings emphasize that without adequate support systems, increased maternal work intensity in China may compromise child health and exacerbate early-life inequalities.

Overall, while parental labor intensity represents a relevant mechanism linking trade liberalization to child health, its effects differ significantly by gender, reinforcing the importance of considering household-level caregiving dynamics in health policy and labor market research.

### 6.4 Left-behind children

Finally, this paper also investigates whether the left-behind status of children mediates the relationship between export shocks and child morbidity, with a specific focus on gender differences. Following Li and Zang (66), a left-behind child is identified as a child residing in a rural area whose father or mother is absent from home due to labor migration, excluding reasons such as divorce, death, or other permanent separations. In addition, this paper further explores gender differences in the mediating effects of left-behind status from a gender-based perspective.

As shown in Table 7, the results from Probit (2) and Probit (5) suggest that export shocks significantly increase the likelihood of children being left behind, especially among girls. However, only the coefficient for female children is statistically significant at the 5% level, while the corresponding coefficient for male children is positive but not statistically significant. These findings imply that girls may be disproportionately affected by labor migration patterns associated with trade expansion, possibly due to prevailing gender roles and caregiving expectations within households.

**Table 7:** Mediation effects: left-behind children.

Variables	Female			Male		
	Probit (1)	Probit (2)	Probit (3)	Probit (4)	Probit (5)	Probit (6)
Export shock	−0.14* (0.07)	0.13** (0.12)	−0.01 (0.10)	−0.14* (0.07)	0.11 (0.11)	−0.23** (0.10)
Individual controls	Y	Y	Y	Y	Y	Y
Household controls	Y	Y	Y	Y	Y	Y
Parental Controls	Y	Y	Y	Y	Y	Y
Community FE	Y	Y	Y	Y	Y	Y
Year FE	Y	Y	Y	Y	Y	Y
Observations	2,867	872	1,343	2,867	1,026	1,553
R-squared	0.04	0.15	0.05	0.04	0.14	0.07

All regressions include a constant term. Robust standard errors are reported in parentheses. All specifications control for individual-, household-, and parental-level covariates, as well as community and year fixed effects. Probit (1–3) and probit (4–6) present the three steps of this mediating effect of left-behind children from different gender perspectives, respectively. Statistical significance is denoted by *p < 0.10, **p < 0.05, and ***p < 0.01.

In the third stage of the mediation model, Probit (3) and Probit (6) assess the relationship between export shocks and child morbidity, conditional on left-behind status. Results indicate that export shocks are significantly associated with a reduction in morbidity among male left-behind children (at the 5% significance level), while the effect is statistically insignificant for female children. This gender-specific outcome may reflect a differential adaptation process or coping capacity between boys and girls in the absence of parental care.

From a theoretical perspective, the impact of being left behind is likely mediated by both resource availability and caregiving quality. In some rural Chinese households, the outmigration of parents may lead to increased remittances and household income, which can improve living conditions and access to healthcare—particularly benefiting boys, who often receive preferential treatment under traditional son-preference norms (67). In contrast, prolonged maternal absence due to labor migration may undermine children’s emotional well-being and reduce the consistency of daily health monitoring, with particularly pronounced adverse effects observed among girls (68).

These findings highlight the nuanced and gendered implications of parental labor migration triggered by trade shocks. As China continues to experience large-scale internal migration, left-behind children remain a vulnerable group that warrants targeted social protection and health policy attention. Addressing the unique needs of female left-behind children, through improved local services, education, and mental health support, should be a priority for reducing health inequalities in rural areas.

## 7 Conclusion

Health is a fundamental and enduring pursuit of human society, and its importance is universally recognized. In the context of China’s diminishing demographic dividend, accelerating population aging, and persistently declining birth rates, children’s health has become increasingly vital for the country’s future economic and social development. This study utilizes data from the China Health and Nutrition Survey (CHNS) and the UN Comtrade Database, and employs a Bartik IV strategy to construct a measure of export shocks while mitigating endogeneity concerns. Based on this framework, we estimate the effect of trade liberalization on child health outcomes in China. The main findings of this study are as follows:

Baseline regression results suggest that export shocks are associated with a significant reduction in child morbidity. Specifically, a one-standard-deviation increase in export exposure corresponds to a 14% decrease in the probability of child morbidity.

Further mediation analysis reveals that export shocks influence child health through four primary channels: parental fixed income, employment rate, work intensity, and the incidence of left-behind children.

For parental fixed income, the mediation model results show that export shocks are associated with increased wages for both fathers and mothers, thereby reducing child morbidity.

In terms of employment, higher paternal employment rates induced by export shocks are linked to improved child health outcomes.

Regarding work intensity, greater paternal work intensity is associated with lower child morbidity, whereas increased maternal work intensity appears to raise child morbidity; however, this latter effect is not statistically significant.

With respect to left-behind children, export shocks significantly increase the proportion of left-behind children in China. However, the mediating effect of left-behind status differs by gender: export shocks are significantly associated with lower morbidity among male left-behind children, while the effect for females is not statistically significant.

Heterogeneity analysis indicates that the association between export shocks and child morbidity varies across subpopulations. The effect is stronger for boys, urban children, and children residing in western China, suggesting that trade exposure does not affect all groups equally.

Effectively mitigating the depletion of health capital is crucial for maintaining competitiveness in the process of international trade development. Based on the empirical findings, this study provides the following policy recommendations:

Firstly, policymakers should strengthen corporate social responsibility oversight and ensure the protection of workers’ legal rights. Employees are the backbone of enterprise development, and businesses should implement measures to safeguard workers’ economic wellbeing while improving workplace conditions and protecting their health.

Secondly, local governments should actively guide regional industries—especially those in rural areas—to create more job opportunities for women, ensuring stable female employment. By increasing women’s employment rates and wages, child morbidity rates can be reduced. At the same time, higher local employment opportunities for women can mitigate the increase in left-behind children caused by parental labor migration, thereby preventing potential adverse health effects. Policies such as tax reductions and subsidies should be introduced to encourage enterprises to provide more job opportunities for women. This approach not only enhances female workers’ economic wellbeing but also enables them to better balance work and family responsibilities, ultimately contributing to improved child health outcomes.

Finally, in the context of ongoing structural reforms, China should further enhance its public health and education service systems while advancing urbanization. Narrowing the disparities in healthcare and education services between urban and rural areas, as well as across regions, is essential to meeting the growing public service demands of the population. Achieving the “Healthy China” strategic goal requires continuous investment in public health infrastructure and policy support. Moreover, improving public services is an effective way to mitigate the depletion of health capital during trade-driven economic growth, ensuring a healthier future for the next generation.

While this study contributes to the literature, it also has limitations, and several important questions remain open for future research. The first is related to the identification strategy. Although the Bartik-IV approach enhances causal inference by addressing potential endogeneity, it remains an observational method that relies on the assumption that national-level export shocks are exogenous to local health outcomes, conditional on baseline industry structure. This assumption may be challenged if certain industries are heavily concentrated in specific regions, thereby weakening the exclusion restriction. Future research could further assess the validity of the instrument by incorporating alternative identification strategies or conducting additional falsification tests. The second question that requires more research is the potential role of environmental transmission mechanisms. While this study focuses on the overall association between export shocks and child health, it does not explicitly examine the environmental consequences of export expansion. As China’s export activities continue to grow, trade-related pollution and ecological degradation are likely to have indirect yet significant effects on child health. Empirical studies that explore how such environmental externalities mediate the relationship between trade and health would be valuable. Investigating these two issues would help to advance a more comprehensive understanding of the long-term social and environmental costs of globalization, particularly for vulnerable populations such as children. Finally, although the CHNS dataset captures substantial heterogeneity across multiple provinces in China, it is not strictly representative of the entire national population. Therefore, while the findings offer important insights into the relationship between trade liberalization and child health, their generalization beyond the sampled regions should be interpreted with caution.

## Data Availability

The datasets presented in this study can be found in online repositories. The names of the repository/repositories and accession number(s) can be found in the article/supplementary material.

## Footnotes

^1^Data source: U.S. Department of Commerce. https://2017-2021.commerce.gov/news/press-releases/2020/12/commerce-department-will-publish-first-military-end-user-list-naming.html (Date of application: 15.01.2025).

^2^Data source: General Administration of Customs of the People’s Republic of China, https://www.customs.gov.cn/customs/syx/index.html (Date of application: 20.01.2025).

^3^While the CHNS data provides rich micro-level information and captures substantial heterogeneity across key Chinese provinces, it does not claim strict national representativeness. Therefore, our estimates should be interpreted as indicative of effects in broadly representative regions, rather than definitive for the entire national population.

^4^UN comtrade database: the database is the world’s most comprehensive global trade data platform. The United Nations Commodity Trade Statistics Database aggregates detailed annual and monthly global trade statistics by product and trading partner. The data cover approximately 200 countries, accounting for more than 99% of global merchandise trade.

^5^Explanation of the variable Exports Change: combined with the UN Comtrade Database, expressed as the difference between period t and period t-1.

^6^Child morbidity is measured based on a self-reported four-week recall question from the CHNS: “In the past 4 weeks, have you been ill or injured?” While the data reflects a short recall period, the survey’s randomized sampling within each wave and large coverage allow this measure to reasonably approximate annual child health conditions.

^7^Although Bartic-IV mitigates endogeneity concerns and enhances causal inference, it remains an observational method, and the possibility of omitted variable bias cannot be fully excluded.
